# Experience modulates an insect’s response to anthropogenic noise

**DOI:** 10.1093/beheco/arz159

**Published:** 2019-09-27

**Authors:** Mario Gallego-Abenza, Nicolas Mathevon, David Wheatcroft

**Affiliations:** 1 Konrad Lorenz Forschungsstelle, Core Facility for Behaviour and Cognition, University of Vienna, Austria; 2 Department of Cognitive Biology, University of Vienna, Vienna, Austria; 3 Equipe Neuro-Ethologie Sensorielle, ENES/Neuro-PSI CNRS UMR9197, University of Lyon, Saint-Etienne, France; 4 Department of Ecology and Genetics, Uppsala University, Uppsala, Sweden

**Keywords:** acoustic adaptation, anthropogenic noise, behavioral plasticity, *Gryllus bimaculatus*, insect, sexual signals

## Abstract

In response to anthropogenic noise, vertebrates express modified acoustic communication signals either through individual plasticity or local population adaptation. In contrast, how insects respond to this stressor is poorly studied. Field crickets *Gryllus bimaculatus* use acoustic signals to attract and locate mates and are commonly found in noisy roadside environments, offering a powerful system to study the effects of anthropogenic noise on insect communication. Rapid repetition of sexual calls (chirps) is essential to attract females, but calling incurs energetic costs and attracts predators. As a result, males are predicted to reduce calling rates when background noise is high. Here, we combine observations and experimental playbacks to show that the responses of field cricket males to anthropogenic noise also depend on their previous experience with passing cars. First, we show that males living on highway edges decrease their chirp rate in response to passing cars. To assess whether this behavioral response depends on previous exposure to car noise, we then broadcast recordings of car noise to males located at different distances from the road and, therefore, with different previous exposure to car noise. Although all tested individuals responded to broadcasted traffic noise, males closest to the road decreased their chirp rate less than individuals calling further from the road. These results suggest that regular exposure to anthropogenic noise may decrease individuals’ sensitivity and behavioral responses to noise, allowing them to maintain effective signaling rates. Behavioral plasticity modulated by experience may thus allow some insect species to cope with human-induced environmental stressors.

## Teaser

Field crickets modulate their acoustic signals based on their degree of previous experience with traffic noise, which may help them avoid its interfering effects. These results help us understand how insects living in urban areas accommodate novel abiotic factors, in particular, traffic noise created by humans.

## INTRODUCTION

Signaling in noisy environments can reduce perception by intended receivers, leading animals to flexibly adapt their signals in response to natural variation in noise. For example, the presence of signaling con- and hetero-specifics (Ryan and Brenowitz 1985; Bleach et al. 2015), moving bodies of water (Feng et al. 2006), wind (Lengagne et al. 1999), or dense foliage (Mathevon 2005) influence animals’ acoustic communication behavior. In addition to natural biotic and abiotic sounds, noise created by human activities—anthropogenic noise—has been increasingly understood to exert strong selection on animals’ acoustic communication signals. For example, anthropogenic noise is often loudest at low frequencies, and individuals from some anurans, insects, and songbird species shift the signal band upwards to avoid masking (Slabbekoorn and Peet 2003; Parris et al. 2009; Lampe et al. 2012; Potvin and Mulder 2013; Slabbekoorn 2013; but see Brumm and Bee 2016; Brumm 2017). Another strategy to improve the signal-to-noise ratio consists of increasing the signal amplitude, the *Lombard effect* (Lombard 1911), which has been demonstrated in, for example, male “nightingales, *Luscinia megarhynchos*” (Brumm 2004). Both shifting frequency and increasing amplitude have been described to co-occur as-side effects in “common blackbirds, *Turdus merula*” singing in noisy environments due potentially to vocal constrains (Nemeth et al. 2013). Finally, individuals from some species reduce or alter their acoustic activity when noise is present, which has been described in songbirds (Ficken et al. 1974; Fuller et al. 2007; Díaz et al. 2011) and mammals (Egnor et al. 2007). Given that human-made noise varies both spatially and temporally, an important distinction is the degree to which responses to noise are influenced by previous exposure, either on the individual or population level.

Individuals songbirds, for example, the “silvereye, *Zosterops laterallis*” from both rural and urban areas may similarly lower their call minimum frequency when exposed to high-frequency noise (Potvin and Mulder 2013), suggesting that adaptive responses to anthropogenic noise arise through general behavioral plasticity irrespective of previous exposure to noise. However, long-term exposure over evolutionary timescales may also generate local adaptation. A recent study conducted in “great tits, *Parus major*” pointed out the effect of noise exposure over generations at the population level as the main force behind frequency shifts in song (Zollinger et al. 2017). They experimentally showed no noise-induced frequency shifts in song by exposing individuals to chronic noise during both vocal learning and in adult stages (i.e., after song crystallization). Finally, previous exposure to noise can itself alter the strength of individual reaction norms, which may offer a powerful strategy to adjust communication behavior, particularly when anthropogenic noise varies greatly over both short spatial and temporal scales. For example, LaZerte et al. (2016) reported that “black-capped chickadee, *Poecile atricapillus*” individual’s vocal response to experimental noise is modulated by its prior experience with anthropogenic noise.

Responses to anthropogenic noise have been well studied in vertebrates, including songbirds, but acoustic communication is vital for territorial and mating interactions in insects as well (Hoy and Robert 1996). As a result, anthropogenic noise may have important consequences both at the population and individual levels (Wilson et al. 1999), and, because insects comprise a fundamental part of ecosystems, also at the ecosystem scale (Morley et al. 2014). For instance, arthropod species have been shown to drastically decrease in abundance in noisy compared with quiet areas (Bunkley et al. 2017). Insects are known to develop evolutionary adaptations or short-term behavioral modifications to cope with natural sources of noise. For instance, the cricket *Paroecanthus podagrosus*, which lives in noisy rainforests, has developed a sharply tuned auditory system that excludes other environmental sounds, which differs from the broader tuned system of its European counterparts (*Gryllus bimaculatus* and *Gryllus campestris*) living in quieter areas (Schmidt et al. 2011).

In spite of the fact that the broadly tuned auditory systems of many insect species include the frequencies in which anthropogenic noise is loudest (Hoy and Robert 1996; Morley et al. 2014), only a few studies have investigated how insects respond to variation of anthropogenic noise at the individual or population level. For instance, it had been shown that “bow-winged grasshopper, *Chorthippus biguttulus*” males signaling near noisy roads produce songs with higher carrier frequencies than those from quiet areas (Lampe et al. 2012). Similarly, cicadas *Cryptotympana takasagona*, singing in noisy environments have been reported to shift their songs to higher frequencies (Shieh et al. 2012). Calling or not might be also present as an insect-specific response to traffic noise; the “tree cricket, *Oecanthus pellucens*” decreases signaling effort when high levels of noise occur (Orci et al. 2016). Negative fitness consequences due to previous exposure to noise during developmental stages have been demonstrated in field crickets, *Teleogryllus oceanicus*, where exposure to noise during rearing hindered female location of males (Gurule-Small and Tinghitella 2018) or delayed maturity and reduced life span (Gurule-Small and Tinghitella 2019). Finally, Lampe et al. (2014) demonstrated that an exposure to high levels of traffic noise during development induces the grasshoppers *C. biguttulus* males to produce songs composed by higher frequency elements. Aside from this last study, we lack knowledge of whether noise-induced modifications in insect communication behavior arise via experience-independent or experience-dependent individual plasticity.

In the present study, we focus on the calling behavior of the field cricket *G. bimaculatus* to address this question. In this species, sedentary males sing from natural holes in the ground to attract females (Simmons 1986; Jacot et al. 2008), whereas females are known to locate singing males (Schmidt et al. 2014). Individuals are abundant in environments with significant anthropogenic noise, such as the edges of roads and highways, this fact together with their male-specific sedentary behavior make them a perfect model species to study the effect of noise exposure on males’ song. Although the frequency of males’ song (4.7–5.7 kHz; Miyashita et al. 2016) lies above the main frequency bandwidth of tyre-made noise, 0.9–1 kHz, females fail to locate singing males under noisy conditions, which is thought to be due to the broad tuning of their auditory system and the potential distraction created by the noise stimulus (Schmidt et al. 2014). Such hindered female location of males is consistent with the idea that anthropogenic noise may have serious deleterious effects on male–female communication. Females are more attracted to individuals, typically young males, who produce songs with a higher chirp rate (Verburgt et al. 2011). Older males sing at lower chirp rate due to the age-related degradation of stridulatory muscle performance, however, they increase the number of pulses per chirp to compensate (Verburgt et al. 2011). This makes both the chirp rate and pulses per chirp secondary sexual traits to keep stable to transmit honest information to attract females while also coping with fluctuating traffic noise. To investigate the effect of traffic noise on males’ singing behavior, we first recorded singing individuals along highways to assess alterations in both chirp rate and number of pulses per chirp during natural peaks of noise caused by passing cars. We then experimentally broadcast recordings of traffic noise to males located at different distances from the road and thus, with different daily traffic noise experiences. We investigated: 1) if field cricket males decrease their singing activity (in terms of chirp rate and number of pulses per chirp) in response to passing cars and 2) whether the strength of these changes in singing activity depends on individual experience with road noise.

## MATERIALS AND METHODS

### Study area and species

**Figure 1:**
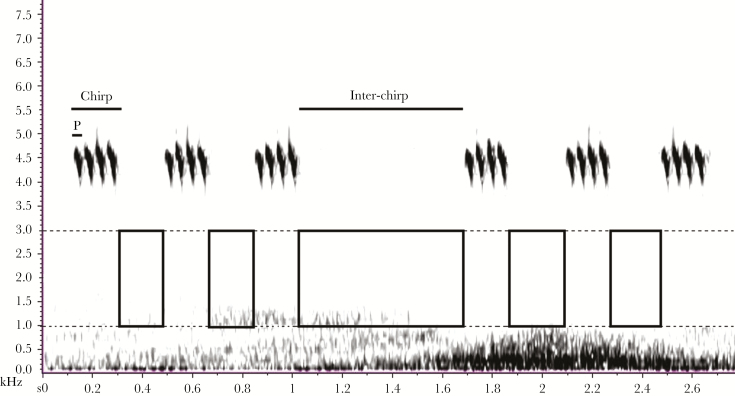
Spectrogram on Raven Pro 1.5 software showing 1) *Gryllus bimaculatus* male song and 2) how both noise intensity and inter-chirp duration measurements were taken simultaneously, and the number of pulses (“P”) per chirp was counted.

The field cricket *G. bimaculatus* (De Geer 1773) has a wide global distribution (Mediterranean regions of France and Spain, Madagascar, Morocco, Ethiopia, and central Asia; Défaut, 1999). Males’ calling song consists of a sequence of chirps composed of 3–5 pulses (Figure 1). Field recordings and playback experiments were conducted in the southern Spain, Region of Murcia during the years 2016 and 2018.

### Field observations

In early May 2016 (2nd and 4th), we recorded between 1 and 4 min of the singing activity of 10 individual males singing along a highway edges (RM-19: 37°51′41″N, 1°5′8″W), at 3–5 m from the sound source. To limit disturbances due to approaching the animals, we used a shotgun directional microphone (Sennheiser ME67) plugged into a Tascam DR-100mkII (sampling rate = 44.1 KHz, 16-bits, WAV format). After recording, we checked for species-specific morphological features of adult males, such as the size of the head compared with the pronotum and the developmental shape of wings, to confirm species identity.

### Assessment of noise gradient

**Figure 2:**
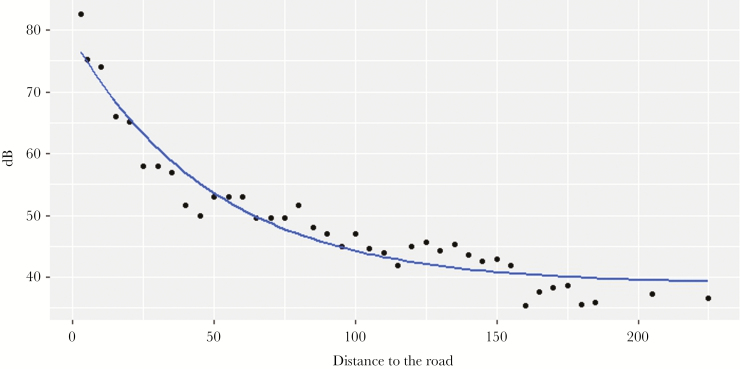
Exponential decay relationship between the loudness of a passing car (dB) and distance to the road (meters).

To assess the gradient of road noise (the attenuation in noise intensity in function of distance to road), we measured the amplitude of peaks of noise produced by three different passing cars at different distances to the highway: 3 m and every 5 m from 5 to 185, at 205 and 225 m of distance. We found that car noise decayed to nearly background levels at 185 m, thus, we took measurements every 20 m from this point (see Figure 2 showing the noise-distance gradient). We used a digital sound level meter (RadioShack model 3300099, A weighting, fast response) held up above the ground and oriented toward the road. We performed these measurements on a flat area, on the side of a highway where background noise (without passing cars) was 34–36 dB. We used the function “Self-Starting Nls Asymptotic Regression Model” in the “nlstools package” (Baty et al. 2015) to fit the exponential decay curve which describes the noise-distance gradient (Figure 2). Once calculated “Asym,” “R0” and “lrc” parameters, the following formula was used to estimate the noise intensity (in dB) “Baseline Car dB” at which crickets would be exposed to depending on their distance to the road (“input” in the formula).

$$\begin{array}{l} Baseline\;Car\;dB=Asym+\left(R0-Asym\right)\times e^{\left(-e^{\left(lrc\right)}\times input\right)} \end{array}$$

### Preparation of road noise playbacks

We recorded traffic noise in an open area, at a distance of 4 m from the road using a digital voice recorder (Olympus DM650, frequency range = 40 Hz–21 kHz, sampling rate= 44.1 Hz, 16-bit, WAV format). We used Raven Pro 1.5 software (Cornell Lab of Ornithology, Ithaca, NY) to isolate five frames of 90 s each, containing between five and eight independent passing cars each. We checked all recordings through spectrogram inspection to ensure that they do not contain any additional sound. Before the playback experiments, we used the sound level meter described above to measure the peaks of noise from playback of cars and adjust thus the volume to mimic the same sound pressure level (75–81 dB) that real passing cars produce when measured at 4–5 m to the road.

### Playback experiments

In April 2018 (18th–21st), we conducted playback experiments on 22 males located at different distances (distance to road range = 7–1446 m) from three roads (RM-19: 37°51′41″N, 1°5′8″W; RM-F19: 37°53′14″N, 0°59′45″W; RM-1: 37°54′40″N, 0°57′28″W) and thus exposed to different levels of noise consequently. We chose four nights with no wind and similar temperature (16–19 °C). The playback experiments were performed between 2300 and 0420 PM. We assessed the exact GPS position of each tested animal using the “UTM GEO MAP” application on the smartphone BQ Aquaris U Plus. Distances to the road were measured in Google Earth© using the built-in meter tool.

### Experimental protocol

Before playback, we placed a tripod holding a Bluetooth loudspeaker (JBL Flip 4, frequency response 70–20 000 Hz) at 55 cm above the singing male, positioned such that the playback stimulus came from the same direction of the road. We placed a digital voice recorder (Olympus DM650) on the ground pointing at it at a distance of 60 cm from the focal. We then waited for the cricket to resume singing for at least two continuous minutes before presenting the noise treatment. The noise treatment was then broadcast using a mobile smartphone (BQ Aquaris U Plus) connected to the Bluetooth loudspeaker. Each male was tested once. We randomized the order of noise treatments and trials interrupted by passing cars were not included in the analyses.

### Acoustic analysis of singing behavior

Recordings (sampling rate= 44.1 Hz, 16-bit, WAV format) were analyzed by MGA and using Raven 1.5 Pro (Cornell Lab of Ornithology). For the observational analysis, we randomly chose 30 consecutive inter-chirp measurements from each male, involving at least two independent passing cars. For the playback experiments, we analyzed the behavioral response of each tested male during the 90 s of noise treatment from the recording, during which calling song and played back car noise co-occur. In both cases, we used consistent spectrogram settings (FFT size = 512, window function = Hann, frequency resolution of 124 Hz, and temporal resolution of 11.6 ms). First, to associate inter-chirp duration with noise, we measured each inter-chirp interval (in seconds, using “Delta time” function) and the amplitude of noise contained between 1 and 3 kHz ["Max Power dB"] in that interval. Resulting dB in “Max Power dB” refers to “Observational car dB” and “Experimental car dB” in the observational and experimental parts of this study, respectively. Second, to associate pulses per chirp with noise, for each inter-chirp interval, we counted the number of pulses per chirp for the chirp immediately following the interval (Figure 1).

Following Lampe et al. (2012), we also measured the peak of frequency of our tested cricket males song. We isolated five chirps per individual before the noise treatment occurred. We used the function “fpeaks” in the package “seewave” (Sueur et al. 2008) to both detect and extract the peak of frequency contained in each chirp.

### Statistical analyses

We conducted statistical analyses within the R framework (version 3.4.1., R Core Team 2014). For the inter-chirp duration, we used the function lmer in the package “lme4” (Bates et al. 2015), and, for the number of pulses per chirp, we used the function clmm, cumulative link mixed model, in the “ordinal” package (Christensen 2018). We included “Observational car dB” and “Experimental car dB” (representing noise amplitude in observational and experimental parts, respectively) as fixed effects and the identity of the recorded male as a random effect (*N* = 10 for observational and *N* = 22 for the experimental part). For the playback experiment analyses, we included two additional random variables: “treatment” (*N* = 5) and “road” (*N* = 3). To assess whether the interaction between “Experimental car dB” and “Baseline car dB” could explain the relationship between the behavioral response and the noise level exposed in tested males, we ran four models: the null model, “Experimental car dB,” “Experimental car dB” + “Baseline car dB,” and “Experimental car dB” × “Baseline car dB.” We based our model selection in AIC values (Burnham and Anderson 2002) to determine which model explained the most variation (Table 1).

**Table 1:** Summary of linear mixed-effects models showing how (a) inter-chirp interval and (b) number of pulses per chirp were adjusted according to played back noise [Experimental car (dB) and Baseline car (dB)] during playback experiments

Model	Df	Log likelihood	AIC	∆AIC	
a) Inter-chirp interval					
Experimental car (dB) × Baseline car (dB)	8	−6170.6	12357.2	0.0	
Experimental car (dB)	6	−6177.4	12366.8	9.6	
Experimental car (dB) + Baseline car (dB)	7	−6177	12368.0	10.8	
Null	5	−6246.6	12503.3	146.1	
**Selected model**	**Parameter**	**Estimate ± SE**	**df**	***t***	***P***
Experimental car (dB) × Baseline car (dB)	Intercept	−2.545 ± 0.978	33.60	−2.600	0.0137
	Experimental car (dB)	0.086 ± 0.013	2951	6.349	**< 0.001**
	Baseline car (dB)	0.032 ± 0.019	28.60	1.649	0.110
	Experimental car (dB) *×* Baseline car (dB)	−0.0009 ± 0.0002	2953	−3.585	**< 0.001**
	**df**	**Log likelihood**	**AIC**	**∆AIC**	
b) Pulses per chirp					
Experimental car (dB) × Baseline car (dB)	11	−1226.27	2474.55	0.0	
Experimental car (dB)	9	−1228.38	2474.76	0.2	
Experimental car (dB) + Baseline car (dB)	10	−1228.18	2476.36	1.8	
Null	8	−1250.18	2516.36	41.8	
**Selected model**	**Parameter**	**Estimate ± SE**	***z***	***P***	
Experimental car (dB) × Baseline car (dB)	—				
	Experimental car (dB)	−0.0784 ± 0.0226	−3.459	**0.0005**	
	Baseline car (dB)	−0.0004 ± 0.0645	−0.007	0.9941	
	Experimental car (dB) × Baseline car (dB)	0.0008 ± 0.0004	1.962	**0.0497**	

Finally, we used linear mixed model, function lmer to investigate whether the daily noise exposure “Baseline car dB” had an effect on the peak frequency of the chirps, using “road” as a random effect.

## RESULTS

### Observational data

Inter-chirp duration was positively associated with “Observational car dB” produced by passing cars (Estimate = 0.005, SE = 0.001, df = 238.81, t = 5.11, *P* < 0.001). This translates into a decrease of chirp rate when a car passes (Table 1). “Observational car dB” had no significant effect on the number of pulses per chirp (Estimate = −0.037, SE = 0.031, *z* = 1.174, *P* = 0.24).

### Playback experiments

**Figure 3:**
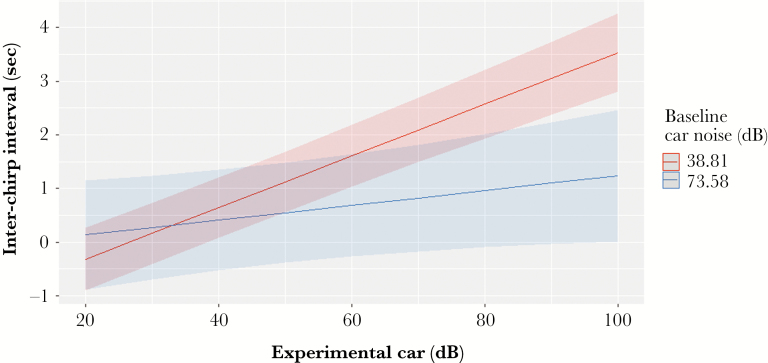
Predicted values for “Inter-chirp interval duration (sec)” responses to played back car noise “Experimental car (dB)” based on reported min and max values of the “Baseline car noise (dB).” The interaction between the two factors predicts crickets from quieter areas to increase Inter-chirp interval significantly more than those exposed to higher levels of car noise.

In the line with our observational results, we found a significant positive effect of the experimental broadcasted noise “Experimental car dB” on the inter-chirp duration (Estimate = 0.0389, SE = 0.0032, df = 2945, *t* = 11.97, *P* < 0.001). Moreover, we found a significant interaction between “Experimental car dB” and the “Baseline car dB” (see Table 1 for model comparison based on AIC values): individuals at closer distances to the road reduced their chirp rate less than individuals further away from the road in response to a given noise (Figure 3). We found a similar significant interaction between “Experimental car dB” and “Baseline car dB” on the number of pulses per chirp (Table 1): individuals located close to the road tended to decrease the number of pulses per chirp significantly less than those located far from the road (Table 1). We did not find a significant relationship between “Baseline car dB” and the frequency peak of the chirp (Estimate = −0.0001, SE = 0.0009, *t* = 0.131, *P* = 0.89).

## DISCUSSION

Our results demonstrate that field cricket *G. bimaculatus* males adjust their singing behavior to match the current level of ambient noise; they rapidly decrease their chirp rate in response to louder traffic noise. Moreover, the magnitude of a male’s response to broadcast car noise depends on its current distance to the road, with individuals closer to the road having significantly reduced responses in chirp rate and maintaining stable the number of pulses per chirp. Here, we discuss both the mechanism and selective pressure giving rise to this spatial variation in responses to car noise.

Our experimental results show that individuals nearby roads maintain higher chirp rates when exposed to car noise. These findings could be consistent with the hypothesis that an individual’s behavioral response to car noise depends on its previous experience, but there are at least two alternative explanations. First, spatial variation in car noise may have driven local adaptation at micro-geographic scales. This explanation requires changes in population genetic structure over short spatial scales. A genetic study conducted on a sister species *G. campestris* shows very weak population structure in a wild population (Bretman et al. 2011). We studied a population of individuals continuously distributed, making it likely that there is significant exchange of genes between individuals breeding close to and far from roads, which is likely to erode local adaptation over such short spatial scales. The second alternative hypothesis is that individuals with weaker responses to passing cars are more likely to disperse to and establish territories near roadsides, thereby explaining our experimental results. However, previous studies have demonstrated that *G. bimaculatus* males are largely sedentary (Simmons 1986; Schmidt et al. 2014), males of the closely related species *G. campestris* are known to move over very short spatial scales, on the order of 9 m on average (Fisher et al. 2016). In contrast, our experiments were conducted over relatively large spatial scales, on the order of 7–1400 m to the road, making it unlikely that individuals spatially sort themselves according to their natural acoustic responses to noise. As a result, although future manipulative experiments could definitively demonstrate a role for individual experience, we conclude that local adaptation and individual dispersal are unlikely explanations for our findings.

Individual experience could influence adult acoustic behavior through a number of mechanisms. First, habituation to noise may be a result of early imprinting. For example, Lampe et al. (2014) showed that noise exposure during developmental stages could explain differences in acoustic properties of adult songs in *C. biguttulus.* Second, it is possible that field crickets maintain their ability to learn over the adult age. Future research on could experimentally disentangle which period of life of *G. bimaculatus* males may play a role in imprinting to road noise.

The broad tuning of the cricket auditory system suggests that car noise interferes with female perception of male song (Schmidt et al. 2014). As a result, males might be expected to reduce their chirp rate during noisy periods because females would be unable to locate them anyway. However, an alternative explanation is that male crickets naturally associate car noise with an approaching predator and reduce their chirp rate as part of their general responses to perceiving nearby predators. For example, *Gryllus integer* males stop calling when they perceive a predator nearby (Hedrick 2000). In this case, males calling nearby roads may have learned, through repeated exposure, to disassociate car noise with a predator and, thereby, maintain higher calling rates when cars pass. In contrast, males further from the road and with reduced exposure may express natural anti-predator responses, taking longer pauses to resume calling when a car passes, as shown in Figure 3.

No matter the driver of differences among males singing at different distances to roads, we highlight the potential fitness consequences of maintaining higher chirp rates. It is known that *G. bimaculatus* females are more attracted by young males that sing with higher chirp rates, leading chirp rate to be considered as an honest sexual signal (Verburgt et al. 2011). In addition, males increase chirp rate in the presence of competitors (Lyons and Rnard 2006). Our results show that males living in noisy areas dynamically decrease their chirp rate while cars are passing. But, they also demonstrate that these males keep their number of pulses per chirp more stable than males living in quieter areas. Individual males may have developed a tolerance to noise under the constraints of sexual selection: the need for attracting females would have led males to not over-reduce their chirp rate and, thereby, profit from the intermittent periods of silence in between passing cars to send intact signals. Thus, experienced males may produce undisrupted signals, while efficiently reducing singing effort when signals are unlikely to be transmitted. Such a balance between the benefits of signal transmission and the costs of signaling are similarly reflected in the recent findings on “Túngara frog, *Physalaemus pustulosus*” males, which adapt their sexual displays in urban environments under both sexual and natural selection (Halfwerk et al. 2019).

Further research on calling song ontogeny and controlled periods of noise exposure might highlight which acoustic characteristic of traffic noise are can be learned by crickets and explain the modifications found in this study. The observed decrease of insect diversity in areas with anthropogenic noise (Bunkley et al. 2017) has suggested the hypothesis that noise alone might prove detrimental to insect population. However, our study provides vital support for the idea that insects have the ability to accommodate anthropogenic noise at the individual level and, therefore, raise the possibility that additional factors associated with urbanization may explain its negative effects on insect populations.

## FUNDING

M.G.A. is funded by Austrian Science Fund (FWF) and the University of Vienna. N.M. is funded by the University of Lyon/Saint-Etienne, the CNRS, the Institut universitaire de France, and the Labex CeLyA.

## Notes

We thank O. Moreno-Valtuille for help collecting data in the field. We also thank the anonymous reviewers for their constructive comments on an earlier version of this article.

Data accessibility: Analyses reported in this article can be reproduced using the data provided by Gallego-Abenza et al. (2019).
